# Heterozygosity for E292V in *ABCA3*, lung function and COPD in 64,000 individuals

**DOI:** 10.1186/1465-9921-13-67

**Published:** 2012-08-06

**Authors:** Marie Bækvad-Hansen, Børge G Nordestgaard, Morten Dahl

**Affiliations:** 1 Department of Clinical Biochemistry, Herlev Hospital, Copenhagen University Hospital, Copenhagen, Denmark; 2 Copenhagen City Heart Study, Bispebjerg Hospital, Copenhagen University Hospital, Copenhagen, Denmark; 3 Department of Clinical Biochemistry, Rigshospitalet, Copenhagen University Hospital, Blegdamsvej 9, Copenhagen, DK-2100, Denmark; 4 Faculty of Health Sciences, University of Copenhagen, Copenhagen, Denmark

**Keywords:** ABCA3, Chronic obstructive pulmonary disease, Genetics, Interstitial lung disease, Surfactant

## Abstract

**Background:**

Mutations in *ATP-binding-cassette-member A3* (*ABCA3*) are related to severe chronic lung disease in neonates and children, but frequency of chronic lung disease due to *ABCA3* mutations in the general population is unknown. We tested the hypothesis that individuals heterozygous for *ABCA3* mutations have reduced lung function and increased risk of COPD in the general population.

**Methods:**

We screened 760 individuals with extreme pulmonary phenotypes and identified three novel (H86Y, A320T, A1086D) and four previously described mutations (E292V, P766S, S1262G, R1474W) in the *ABCA3* gene. We genotyped the entire Copenhagen City Heart study (n = 10,604) to assess the clinical importance of these mutations. To validate our findings we genotyped an additional 54,395 individuals from the Copenhagen General Population Study.

**Results:**

In the Copenhagen City Heart Study individuals heterozygous for E292V had 5% reduced FEV_1_ % predicted compared with noncarriers (t-test: p = 0.008), and an increased odds ratio for COPD of 1.9 (95% CI: 1.1-3.1). In contrast, the A1086D mutation was associated with increased FEV_1_ % predicted (p = 0.03). None of the other *ABCA3* mutations associated with lung function or COPD risk in the Copenhagen City Heart Study. In the larger Copenhagen General Population Study, and in the two studies combined, E292V heterozygotes did not have reduced lung function or increased risk of COPD (p = 0.11-0.98), while this was the case for the positive controls, *surfactant protein-B* 121ins2 heterozygotes and *α*_*1*_*-antitrypsin* ZZ homozygotes.

**Conclusion:**

Our results indicate that partially reduced ABCA3 activity due to E292V is not a major risk factor for reduced lung function and COPD in the general population. This is an important finding as 1.3% in the Danish population has partially reduced ABCA3 function due to E292V.

## Background

Pulmonary surfactant is a thin lipid membrane that covers the alveoli and lowers surface tension, thereby preventing collapse of the alveoli at end-expiration. Pulmonary surfactant consists of phospholipids and proteins mainly synthesized by alveolar type II cells. The pulmonary surfactant is assembled and stored in the alveolar type II cell in lamellar bodies, which are subsequently released to the alveoli by exocytosis [1,2]. The lipid transporter ATP-binding cassette member A3 (ABCA3) is expressed in the limiting membrane of the lamellar bodies. Here it transports several types of lipids making it essential for correct assembly of pulmonary surfactant in the alveolar type II cell [3,4].

Recessive mutations in the *ABCA3* gene have been related to deficiency of pulmonary surfactant leading to neonatal lung disease and chronic lung disease in children [3,5]; however, frequency of chronic lung disease due to *ABCA3* mutations in the general population is unknown. At present more than 150 disease-associated mutations have been identified [3]. A relatively common mutation in *ABCA3*, E292V, is associated with partially impaired ABCA3 function [6] and with milder chronic lung disease in childhood [3,6]. This mutation is situated in a conserved intracellular loop of ABCA3 with importance for ATP hydrolysis activity. The E292V mutation has been found in about 0.3-0.4% of individuals in a heterogeneous US population [7] and may affect both heterozygous [8,9] and compound heterozygous carriers [8,10]. Because mice with 50% reduced ABCA3 die from respiratory distress or develop emphysema [11], individuals heterozygous for *ABCA3* variants may also be at increased risk of emphysema/COPD. Given this, *ABCA3* variants, and in particular E292V, could play an important role on development of common pulmonary disorders in the general population.

In this study we hypothesise that individuals heterozygous for *ABCA3* variants have reduced lung function and increased risk of COPD in the general population. To test this, we first screened 760 adults with extreme lung phenotypes in the Copenhagen City Heart Study to identify *ABCA3* variants that could potentially be associated with lung disease in the general population. Following this we genotyped the entire Copenhagen City Heart Study (n = 10,604) for variants identified by the initial screening to test whether *ABCA3* heterozygotes have reduced lung function or increased risk of COPD. When a positive association was observed, we genotyped an additional population-based study, the Copenhagen General Population Study (n = 54,395) in an attempt to reproduce the finding. As positive controls for reduced lung function due to genetic background, we used *surfactant protein-B* 121ins2 heterozygosity and *α*_*1*_*-antitrypsin* ZZ homozygosity [12,13].

## Methods

### Subjects

The Copenhagen City Heart Study is a prospective general population study of individuals selected based on the Central Population Register Code to reflect the adult Danish population aged 20-80+ years [13,14]. The Copenhagen City Heart Study was initiated in 1976–1978 with follow-up examinations in 1981–1983, 1991–1994, and 2001–2003. DNA was isolated from participants attending the 1991–1994 (n = 9252) and/or 2001–2003 examinations (additional n = 1352).

The Copenhagen General Population Study is an ongoing population-based cohort study initiated in 2003 [12,15]. At the time of genotyping for the present study, 54,395 individuals had been included. Participants were selected based on the Central Population Register Code to reflect the adult Danish population aged 20-80+ years. Participants are recruited from a different part of Copenhagen than the Copenhagen City Heart Study and there is no overlap of individuals between the two studies. The studies were approved by the Danish ethical committees: Nos. KF-100.2039/91 and H-KF-01-421/94. All participants gave written informed consent and all participants were whites of Danish descent.

### Pulmonary endpoints

FEV_1_ and FVC were determined with a dry wedge spirometer (Vitalograph; Maids Moreton, Buckinghamshire, UK) in the Copenhagen City Heart Study, and with EsyOne Spirometer (ndd Medizintechnik, Zurich, Switzerland) in the Copenhagen General Population Study. Algorithms for calculation of FEV_1_ % predicted and FVC% predicted were made using multiple regressions with age and height as covariates on all individuals for men and women separately. COPD was defined as FEV_1_/FVC < 0.7 and FEV_1_ < 80% of predicted (GOLD stages 2–4) [16]; if this definition excluded asthmatics, the results were similar to those presented. To increase the likelihood of detecting genetic variation in *ABCA3* associated with lung disease, we performed resequencing of the *ABCA3* gene in an extreme risk population in the Copenhagen City Heart Study (Additional file 1). The risk population was defined as those individuals with the earliest onset of COPD (n = 175) and asthma (n =174), individuals with interstitial lung disease (n = 31), and individuals with the lowest FEV_1_ % predicted among non smokers (n = 118), lowest FEV_1_ % predicted among smokers (n = 122), and highest FEV_1_ % predicted in the population (n = 140) [17]. We ensured that there was no overlap between the risk groups when selecting these individuals from our database. The number of individuals selected for each extreme phenotype group was based on our laboratory set-up consisting of two 384-well DNA plates (4 wells were for controls). For the first DNA plate we selected all individuals with interstitial lung disease (n = 31). Among the remaining individuals we ranked the patients with COPD according to age and selected the youngest 175 individuals. We then selected the youngest 174 individuals with asthma in the same way making sure that there was no overlap between individuals with interstitial lung disease, asthma, and COPD. For the second DNA plate, we ranked the remaining individuals according to FEV_1_ % predicted and selected those 140 individuals who had the highest FEV_1_ % predicted. We next selected the 122 individuals with the lowest FEV_1_ % predicted among current smokers and the 118 individuals with the lowest FEV_1 _% predicted among exsmokers and nonsmokers to fill out the DNA plate in full.

### Genetic analysis

Thirty PCR fragments were amplified, covering all 30 protein coding exons (exons 4–33) of *ABCA3* and the intron-exon boundaries. Mutational screening analysis of the amplicons was performed by LightScanner, a high resolution DNA melting curve analysis technique for variant detection [18]. PCR fragments with DNA melting curves differing from wild type control DNA were subsequently sequenced. Primer sequences are listed in Additional file 2. We used a TaqMan based assay to genotype the entire Copenhagen City Heart Study for mutations identified by resequencing (Applied Biosystems Inc., Foster City, CA, USA). Primers and probes for these analyses are listed in Additional file 3. Genotype results were confirmed by DNA sequencing of a subset sample. It was not possible to design TaqMan genotyping assays for two of the mutations (A320T and A1086D), and genotyping for these variants was instead performed using the LightScanner (Additional file 3).

### Data analysis

Statistical analyses were performed using STATA version 10.0. A two-sided P < 0.05 was considered significant. Main effects of genotype and statistical interaction between genotype and smoking in predicting FEV_1 _% predicted, FVC % predicted, and FEV_1_/FVC were tested by Student’s t-test or ANCOVA. Odds ratios for spirometry defined COPD according to genotype were determined by a logistic regression model adjusted for age, sex, and packyears of tobacco smoked. As positive controls for reduced lung function and increased COPD risk due to genetic background, we used *surfactant protein-B* 121ins2 heterozygosity and *α*_*1*_*-antitrypsin* ZZ homozygosity [12,13]. We utilized NCSS-PASS (NCSS, Kaysville, UT, USA) to calculate the odds ratios, which we had 80% power to exclude at p-values <0.05.

## Results

Clinical characteristics of the individuals with extreme lung phenotypes are listed in Additional file 1. As expected, individuals with early-onset COPD and interstitial lung disease were older and more likely smokers than individuals without an extreme lung phenotype, whereas individuals with early-onset asthma were younger and less likely smokers [17]. Individuals with the highest or lowest FEV_1_ % predicted did not differ in sex or age from those without an extreme lung phenotype.

### Genetic variation in *ABCA3*

Resequencing of the *ABCA3* gene identified a total of 55 gene variations (Additional file 4). Of these 55 variations, 29 were in protein coding regions and 19 were non-synonymous variants. Seven of the 19 non-synonymous variations changed a polar to a nonpolar aminoacid or vice versa, and thus could be of functional relevance to ABCA3. H86Y, A320T and A1086D were novel variants, whereas E292V, P766S, S1262G and R1474W have been described previously. Two individuals heterozygous for P766S and one individual heterozygous for R1474W suffered from interstitial lung disease (Additional file 4).

A schematic view of the location of the seven variants in relation to the structure of ABCA3 is presented in Additional file 5. The seven gene variants are distributed evenly throughout the protein and are localized to intra- and extracellular loops, the transmembrane helix and the conserved nucleotide binding domain 2, which is important for ABCA3 interaction with ATP [19]. We used the sorting intolerant from tolerant (SIFT) [20] and polymorphism phenotyping (Polyphen) [21] softwares to predict whether any of the seven variants were likely to have a deleterious effect on ABCA3 function. SIFT and Polyphen both predicted E292V to be damaging, but none of the other six mutations.

### Pulmonary function by *ABCA3* genotype in the Copenhagen City Heart Study

We genotyped the entire Copenhagen City Heart Study for all seven *ABCA3* mutations changing a polar to a nonpolar aminoacid, or vice versa. Genotype frequencies and Hardy-Weinberg statistics are listed in Additional file 6. All mutations were in Hardy-Weinberg equilibrium and minor allele frequencies varied from 0.02 % to 0.9%. We calculated D’ and R^2^ and found no linkage disequilibrium between the seven mutations (data not shown).

**Figure 1:**
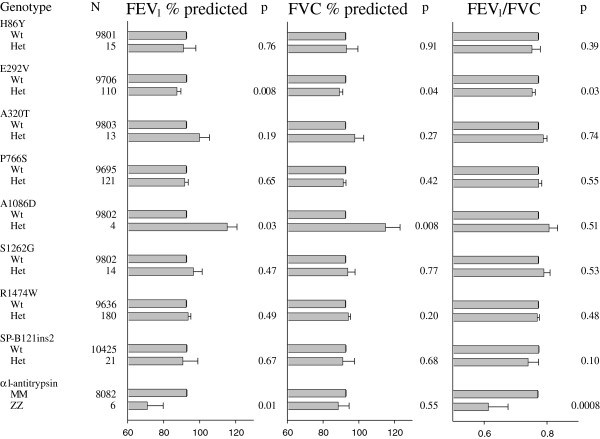
**Lung function according to ATP binding cassette member 3 (ABCA3) genotype in the Copenhagen City Heart Study.** Values are mean and standard error. P-values are by Student’s t-test. Lung function according to *surfactant protein-B* 121ins2 heterozygosity and *α*_*1*_*-antitrysin* ZZ homozygosity serve as positive controls. Numbers of individuals with *surfactant protein-B* 121ins2 heterozygosity and *α*_*1*_*-antitrypsin* ZZ homozygosity differ slightly from the number of individuals with ABCA3 genotypes due to different number of study subjects available for analysis within the study period.

Individuals heterozygous for E292V had 5% reduced FEV_1_ % predicted (t-test:p = 0.008), 3% reduced FVC %predicted (p = 0.04) and 0.02 reduced FEV_1_/FVC (p = 0.03), compared with wildtypes (Figure 1). The observed reductions in pulmonary function were almost similar in absolute numbers to those observed for *surfactant protein-B* 121ins2, a mutation which in the homozygous state associates with a phenotype somewhat similar to that for E292V compound heterozygosity. A 5% decrease in FEV_1_ % predicted would in this study equal a 152 ml reduction of FEV_1_ in a 40 year old female (height 165 cm) or a 214 ml reduction of FEV_1_ in a 40 year old male (height 180 cm).

In contrast, A1086D heterozygotes had increased FEV_1_ % predicted (p = 0.03) and FVC % predicted (p = 0.008) compared with wildtypes, whereas the FEV_1_/FVC ratio showed no statistical significant difference (p = 0.51). Because this result was based on a small number of heterozygous individuals (n = 4), it is likely that this could be a chance finding. Furthermore, due to the improved spirometry observed, this mutation is unlikely to cause lung disease. Thus we did not attempt to reproduce this association in the Copenhagen General Population Study.

Individuals heterozygous for H86Y, A320T, P766S, S1262G, or R1474W did not differ from wildtypes in FEV_1_ % predicted, FVC % predicted or FEV_1_/FVC (p≥0.19). We sought to examine whether a combination of multiple variations in *ABCA3* were associated with reduced lung function and COPD risk; however, only a single individual in the Copenhagen City Heart Study was heterozygous for more than one of the seven variants. This individual was compound heterozygous for P766S and S1262G and was not identified among the extreme phenotypes group. This person had FEV_1_/FVC of 0.71 and FEV1 % predicted of 83%. If correction for multiple comparisons was performed in Figure 1 (P < 0.05/27 comparisons = p < 0.002) only the results for FEV_1_/FVC in ZZ homozygotes would be of statistical significance.

### COPD by *ABCA3* genotype in the Copenhagen City Heart Study

**Figure 2:**
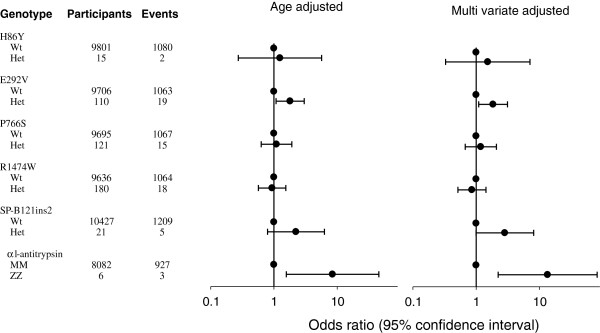
**Risk of COPD according to ATP binding cassette member 3 (ABCA3) genotype in the Copenhagen City Heart Study. **COPD was FEV_1_/FVC < 0.7 and FEV_1_ < 80% of predicted. Values represent odds ratios and 95% confidence intervals. Multivariate adjusted models allowed for age, sex, and packyears. Risk of COPD according to *surfactant protein-B* 121ins2 heterozygosity and *α*_*1*_*-antitrysin* ZZ homozygosity serve as positive controls. Numbers of individuals with *surfactant protein-B* 121ins2 heterozygosity and *α*_*1*_*-antitrypsin* ZZ homozygosity differ slightly from the number of individuals with ABCA3 genotypes due to different number of study subjects available for analysis within the study period.

Individuals heterozygous for E292V had a multivariate adjusted odds ratio for COPD of 1.9 (95% CI 1.1-3.1) compared with wildtypes in the Copenhagen City Heart Study (Figure 2). The observed risk of COPD was slightly lower than that observed for *surfactant protein-B* 121ins2 heterozygotes in Figure 2. For the H86Y, P766S and R1474W mutations, risk of COPD did not deviate significantly from 1.0. This is in accordance with the results on pulmonary function for these variants. Due to lack of events among A320T, A1086D and S1262G heterozygotes, we were not able to calculate risk of COPD for these variants. None of the *ABCA3* mutations associated with asthma in the Copenhagen City Heart Study (data not shown).

### Pulmonary function and COPD by *ABCA3* E292V in the Copenhagen General Population Study

To further validate the findings for E292V, we genotyped the Copenhagen General Population Study (n = 54,395) for the E292V variant. The genotype distribution did not differ from that in the Copenhagen City Heart Study (Additional file 7). Clinical characteristics of individuals from the Copenhagen City Heart Study and Copenhagen General Population Study are displayed in table 1. Characteristics did not differ between E292V heterozygotes and wildtypes in any of the two study cohorts. The fraction of smokers and amount of tobacco smoked were lower in the Copenhagen General Population Study as compared with the Copenhagen City Heart Study [22]. This is likely due to the declining number of smokers in the Danish population over the past decades.

**Table 1:** Characteristics of participants in the Copenhagen City Heart Study and the Copenhagen General Population Study according to E292V genotype

	**Copenhagen City Heart Study**			**Copenhagen General Population Study**		
	**Wildtypes**	**E292V heterozygotes**	**p-value**	**Wildtypes**	**E292V heterozygotes**	**p-value**
N	9,706	110		53,685	710	
Women, %	5,374 (55)	60 (55)	0.86	29,756 (55)	394 (55)	0.97
Age, yrs	58 (44-69)	57 (45-67)	0.57	60 (50-70)	59 (49-69)	0.20
Eversmokers, %	76	77	0.72	60	59	0.40
Packyears of tobacco smoked	25 (11-40)	25 (13-40)	0.72	17 (6-31)	15 (6-30)	0.44

Values represent number, median (interquartile range), or percent. P-values are by Pearson’s χ^2^ test or Wilcoxon rank-sum test.

Results on pulmonary function are displayed in Additional file 8. We found that FEV_1_ % predicted, FVC % predicted, and FEV_1_/FVC did not differ in E292V heterozygotes vs wildtypes (p≥0.67), whereas FEV_1_ % predicted was reduced in the positive controls, *surfactant protein-B* 121ins2 heterozygotes and *α*_*1*_*-antitrypsin* ZZ homozygotes. In accordance with the results on pulmonary function, the multivariate adjusted odds ratio for COPD was not increased in E292V heterozygotes compared with wildtypes (odds ratio 1.10 (0.83-1.46)), while *surfactant protein-B* 121ins2 heterozygotes and *α*_*1*_*-antitrypsin* ZZ homozygotes had increased odds ratios for COPD of 2.8 (1.0-8.1) and 6.6 (1.7-26) (Additional file 9).

### Pulmonary function and COPD by *ABCA3* E292V in the Copenhagen City Heart Study and Copenhagen General Population Study combined

**Figure 3:**
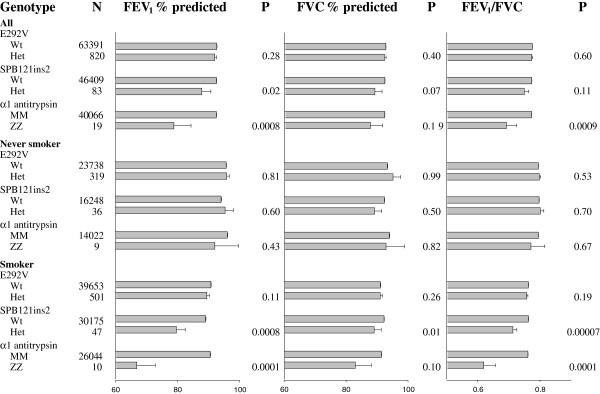
**Lung function according to ATP binding cassette member 3 (ABCA3) E292V, *surfactant protein-B *121ins2, and *α***_***1***_***-antitrypsin*ZZ genotypes in the Copenhagen City Heart Study and Copenhagen General Population Study combined, stratified for smoking status.** Values are mean and standard error. P-values are by Student’s t-test. Lung function according to *surfactant protein-B* 121ins2 heterozygosity and *α*_*1*_*-antitrysin* ZZ homozygosity serve as positive controls. Numbers of individuals with *surfactant protein-B* 121ins2 heterozygosity and *α*_*1*_*-antitrypsin* ZZ homozygosity differ slightly from the number of individuals with an ABCA3 E292V genotype due to different number of study subjects available for analysis within the study period.

Finally, to maximize our statistical power, we combined the Copenhagen City Heart Study and Copenhagen General Population Study. In this analysis, we found that FEV_1_ % predicted, FVC % predicted, and FEV_1_/FVC did not differ in E292V heterozygotes vs wildtypes (p≥0.28), whereas FEV_1_ % predicted was reduced in the positive controls, *surfactant protein-B* 121ins2 heterozygotes and *α*_*1*_*-antitrysin* ZZ homozygotes (Figure 3). We also stratified our data for smoking status, as did our previous study of *surfactant protein-B* 121ins2 as a risk factor in COPD [12], but found no significant differences in lung function for E292V. Among smokers, *surfactant protein-B* 121ins2 heterozygotes had 9% reduced FEV_1_ % predicted (p = 0.0008), 6% reduced FVC % predicted (p = 0.01), and 6% reduced FEV_1_/FVC (p = 0.00007) compared with wildtypes. Corresponding lung function reductions for *α*_*1*_*-antitrypsin* ZZ homozygotes were 24% (p = 0.0001), 8% (p = 0.10), and 19% (p = 0.0001). Among nonsmokers, lung function did not differ in *surfactant protein-B* 121ins2 heterozygotes and *α*_*1*_*-antitrysin* ZZ homozygotes versus wildtypes (p≥0.43).

**Figure 4:**
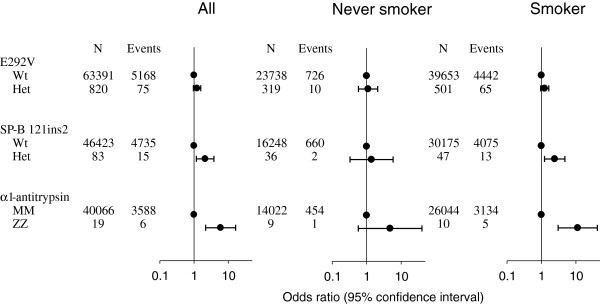
**Risk of COPD according to ATP binding cassette member 3 (ABCA3) E292V, *surfactant protein-B *121ins2, and *α***_***1***_***-antitrypsin*ZZ genotypes in the Copenhagen City Heart Study and Copenhagen General Population Study combined, stratified for smoking status.** COPD was FEV_1_/FVC < 0.7 and FEV_1_ < 80% of predicted. Values represent odds ratios and 95% confidence intervals. Logistic regression models allowed for age, sex, and packyears. Numbers of individuals with *surfactant protein-B* 121ins2 heterozygosity and *α*_*1*_*-antitrypsin* ZZ homozygosity differ slightly from the number of individuals with an ABCA3 E292V genotype due to different number of study subjects available for analysis within the study period.

Individuals heterozygous for E292V had multivariate adjusted odds ratios for COPD of 1.2 (0.96-1.57) among all subjects, of 1.1 (0.6-2.1) among nonsmokers, and of 1.2 (0.95-1.63) among smokers, respectively, compared with wildtypes (Figure 4). Corresponding odds ratios for *surfactant protein-B* 121ins2 heterozygotes were 2.1 (1.7-3.8), 1.4 (0.3-5.9), and 2.4 (1.2-4.8), and for *α*_*1*_*-antitrypsin* ZZ homozygotes 6.0 (2.2-16), 4.8 (0.3-40), and 11 (3.1-42). These results were in accordance with the results observed for lung function for these gene variants. We had 80% power to exclude odds ratios for COPD for E292V heterozygotes of 1.3, for *surfactant protein-B* 121ins2 heterozygotes of 2.1, and for *α*_*1*_*-antitrypsin* ZZ homozygotes of 4.0.

## Discussion

To test whether individuals heterozygous for *ABCA3* variants have reduced lung function and increased risk of COPD in the general population, we screened 760 individuals with extreme lung phenotypes for genetic variations in the *ABCA3* gene and identified three novel (H86Y, A320T, A1086D) and four previously described variations (E292V, P766S, S1262G, R1474W). We next genotyped the entire Copenhagen City Heart Study (n = 10,604) to assess the clinical importance of these seven mutations. We found that E292V heterozygotes had reduced lung function and increased risk of COPD, whereas the novel A1086 mutation was associated with increased lung function. To validate the findings for E292V we genotyped an additional 54,395 individuals from the Copenhagen General Population Study. In this larger cohort, and in the two study cohorts combined, we found that E292V heterozygotes did not have reduced lung function or increased risk of COPD. Supporting this overall negative result, the estimates for the positive controls, *surfactant protein-B* 121ins2 heterozygotes and *α*_*1*_*-antitrypsin* ZZ homozygotes, were relatively stable throughout the studies and there was no evidence of selection bias against E292V in any of the two cohorts.

The phenotype in E292V carriers was previously reported to range from minimal changes on lung biopsy and no symptoms to severe fatal lung disease [8,9]. It is thus possible that E292V may cause lung disease in certain contexts or subgroups of individuals, e.g. when additional impairments of surfactant function are present, leaving the average carrier unaffected. Tobacco smoking [23] and unidentified mutations in *surfactant protein-C*[24,25], *ABCA3*[26], and *surfactant protein-B* could be risk factors of additionally impaired surfactant function in E292V heterozygotes; although the latter risk factors are probably less prevalent in the general population as compared with tobacco smoking. Because participants in the Copenhagen City Heart Study smoked more tobacco than participants from the Copenhagen General Population Study we speculate that the difference in smoking habits between the two cohorts could partly contribute to the different results observed for E292V in the two cohorts. When statistical power, however, was maximised using the Copenhagen General Population Study or the two studies combined, E292V heterozygotes did not differ from wildtypes in lung function or COPD risk. This is an important finding as 1.3% in the Danish general population has partially reduced ABCA3 function due to E292V, and since this variant has been linked previously with severe chronic lung disease in heterozygous and compound heterozygous E292V carriers [8-10].

Besides E292V, we identified six other variants of potential relevance to ABCA3 function. However, none of these mutations were associated with lung function or risk of COPD, except for the novel A1086D mutation. This variant associated with increases in FEV_1_ % predicted and FVC % predicted. However, the small number of A1086D heterozygotes (n = 4) makes this finding highly insecure. In addition, if correction for multiple comparisons was performed none of the results for A1086D and E292V in the Copenhagen City Heart Study would be of statistical significance. The second novel mutation, H86Y, is situated in the first extracellular loop of ABCA3. Another mutation in this loop, L101P, has been shown to affect ABCA3 protein folding leading to retention of ABCA3 in the endoplasmatic reticulum and subsequent ER stress and apoptosis [27,28], however individuals heterozygous for the H86Y mutation appeared asymptomatic in this study. The third novel mutation, A320T, resides in the transmembrane helix domain of ABCA3 and did also not associate with lung function or COPD. Finally, the previously described R1474W mutation may seem particularly interesting as this mutation is situated within the conserved nucleotide binding domain which is important for binding of ATP [19]. However, no association with lung function or risk of COPD was observed for R1474W heterozygosity.

The results of the combined studies showed that *surfactant protein-B* 121ins2 heterozygotes had reduced FEV_1_ % predicted and increased risk of COPD. This observation is novel and extends our previous finding of reduced lung function and elevated COPD risk among 121ins2 heterozygous smokers [12]. Supporting an association between surfactant protein-B and COPD, type II pneumocytes have been proposed to play important roles in COPD development [29]. In line with this, surfactant protein-A [30] and surfactant protein-D [31,32] may be involved in smoking-related lung diseases, and cystic changes and paraseptal emphysema have been previously reported in interstitial lung disease associated with mutations in the surfactant protein-C gene [33,34]. The reduction in lung function and elevated risk of COPD in *surfactant protein-B* 121ins2 heterozygotes in this study was still driven by an effect among smokers, while nonsmokers were unaffected (Figures 3 and 4). Future large epidemiological studies are needed to confirm these results and to further assess whether *surfactant protein-B* 121ins2 combined with other genetic risk factors may be clinically useful in the prediction of COPD. A recent study has demonstrated that combinations of gene variants may associate with up to a 4.7-fold increased COPD risk [35], and many more gene variants related to COPD could be identified and/or implemented in COPD predictions in the future [36-40].

Some degree of misclassification of COPD was possible, since we used prebronchodilator values for lung function to define the disease. However, if the COPD definition excluded asthmatics the results were congruent. All participants in this study are Danish whites and of Danish descent, not reflecting today’s ethnic pattern in the general population. Although this eliminates any blurring due to ethnic heterogeneity of the study population, our results may apply to Caucasians only. Bias caused by investigator knowledge of disease or risk factor status seems unlikely because our sample was selected from the general population and because genotyping of our sample was performed without investigator knowledge of disease status or lung function test results.

## Conclusions

We found with significant statistical power that *ABCA3* E292V heterozygotes do not have reduced lung function or increased risk of COPD in the general population. This is an important finding as 1.3% in the Danish general population has partially reduced ABCA3 function due to E292V and since this variant has been linked previously with severe chronic lung disease in E292V heterozygotes and compound heterozygotes.

## Abbreviations

ABCA3: ATP-binding cassette member A3; COPD: Chronic obstructive pulmonary disease; E292V: Substitution of valine for glutamic acid at aminoacid-position 292; FEV_1_: Forced expiratory volume in one second; FVC: Forced vital capacity.

## Competing interests

The authors declare that they have no competing interests.

## Authors’ contributions

MBH, BGN, and MD were involved in conception, hypothesis delineation, and design of the study. Database handling and statistical analyses were by MBH and MD, while all three authors contributed to analyses and interpretation of the data. MBH wrote the first draft of the paper, which was scrutinized and finally accepted by the other two authors. All authors read and approved the final manuscript.

## Supplementary Material

Additional file 1 **Table S1.** Characteristics of participants with extreme lung phenotypes in the Copenhagen City Heart Study. Click here for file

Additional file 2 **Table S2.** Primers used for resequencing. Click here for file

Additional file 3 **Table S3.** Primers and probes for genotyping assays. Click here for file

Additional file 4 **Table S4.** Genetic variation in the coding regions of*ABCA3* in individuals with extreme lung phenotypes in the Copenhagen City Heart Study. Click here for file

Additional file 5 Figure S1. Click here for file

Additional file 6 **Table S5.** Genotype distribution, minor allele frequency and Hardy-Weinberg statistics for *ABCA3* variants identified in the Copenhagen City Heart Study. Click here for file

Additional file 7 **Table S6.** Genotype distribution and minor allele frequency of ABCA3 E292V in the Copenhagen City Heart Study (CCHS) and the Copenhagen General Population Study (CGPS). Click here for file

Additional file 8 Figure S2. Click here for file

Additional file 9 Figure S3. Click here for file
