# Supplementary material for: Heterozygosity for E292V in ABCA3, lung function and COPD in 64,000 individuals
Source: Respir Res. 2012 Aug 6;13(1):67. doi: 10.1186/1465-9921-13-67 (PMC3514156; doi:10.1186/1465-9921-13-67)
Supplement: Additional file 1 — Table S1. Characteristics of participants with extreme lung phenotypes in the Copenhagen City Heart Study. [file 1465-9921-13-67-S1.doc]

Supplementary table 1. Characteristics of participants with extreme lung phenotypes in the Copenhagen City Heart Study.

|  | No extreme phenotype  (n=9,844) | Asthma  (n=174) | COPD  (n=175) | Interstitial  lung disease  (n=31) | High FEV1  (n=140) | Low FEV1  smokers  (n=122) | Low FEV1  nonsmokers  (n=118) |
| --- | --- | --- | --- | --- | --- | --- | --- |
| Age | 59 (45-70) | 44 (35-53) | 61 (56-66) | 68 (60-74) | 55 (43-66) | 59 (44-68) | 58 (42-68) |
| Male/Female, % | 44/56 | 36/64 | 49/51 | 39/61 | 45/55 | 43/57 | 46/54 |
| BMI | 25 (22-28) | 26 (23-29) | 26 (22-29) | 24 (20-27) | 24 (22-27) | 23 (21-27) | 25 (23-29) |
| FEV1, % predicted | 94 (83-105) | 89 (78-97) | 65 (50-80) | 64 (43-92) | 133 (124-141) | 40 (31-58) | 52 (36-67) |
| FEV1/FVC | 0.79 (0.74-0.83) | 0.79 (0.73-0.83) | 0.67 (0.57-0.75) | 0.70 (0.57-0.81) | 0.82 (0.80-0.87) | 0.54 (0.45-0.69) | 0.63 (0.50-0.78) |
| Current smoker, No (%) | 4,952 (47) | 24 (14) | 144 (83) | 16 (52) | 41 (29) | 122 (100) | 0 (0) |
| Former smoker, No (%) | 3,089 (29) | 77 (44) | 27 (15) | 13 (42) | 46 (33) | 0 (0) | 83 (70) |
| Packyear | 15 (0-35) | 2 (0-15) | 40 (26-53) | 33 (23-48) | 2 (0-17) | 34 (20-44) | 16 (0-38) |

Values represent median (interquartile range) or percent unless otherwise stated. Asthma = yes to the question “do you have asthma?” Chronic obstructive pulmonary disease = FEV_1_/FVC<0.7 and FEV_1_ % predicted < 80%, excl. asthmatics. Interstitial lung disease = ICD8: 517, ICD10: J84. High FEV_1_ = the 140 individuals who had the highest FEV_1_ % predicted. Low FEV_1_ smokers = the 122 individuals with the lowest FEV_1_ % predicted among current smokers. Low FEV_1_ nonsmokers = the 118 individuals with the lowest FEV_1_ % predicted among exsmokers and nonsmokers.
