# Supplementary material for: Heterozygosity for E292V in ABCA3, lung function and COPD in 64,000 individuals
Source: Respir Res. 2012 Aug 6;13(1):67. doi: 10.1186/1465-9921-13-67 (PMC3514156; doi:10.1186/1465-9921-13-67)
Supplement: Additional file 2 — Table S2. Primers used for resequencing. [file 1465-9921-13-67-S2.doc]

| Supplementary table 2. Primers used for resequencing | | | |  | |  |
| --- | --- | --- | --- | --- | --- | --- |
| Fragment | Region | Forward | Reverse | |  | |
| 1 | Exon 4 | AGGGGTGTTTGGAGTTA | AAGAGTTCCAGGACCGT | |  | |
| 2 | Exon 5 | GGAAGGGCTGTCTGCATGC | CAGGTTGAAGTAGTGATGCG | |  | |
| 3 | Exon 6 | CACGCAGATTTTTCAAGCAGG | TGCTATTGACTTGCAGGCAGG | |  | |
| 4 | Exon 7 | GGTGAGTTCTCGGCTGT | TATGTTTATTGCGGCACTA | |  | |
| 5 | Exon 8 | CATGGACCCCATGCACG | TTTGGACATGGCCTCCC | |  | |
| 6 | Exon 9 | ACAGTCGGACTCAGGCC | TCTGACCACAAAGTTCT CC | |  | |
| 7 | Exon 10 | TCCTGGTCCACCTCTGCC | TGGTCAGCTCCTCCCTGG | |  | |
| 8 | Exon 11 | GTGTAGATGGCAAGTGCCAG | GATGCTGCTGCCTTCAGTG | |  | |
| 9 | Exon 12 | CATGCCAACCAAGCAGTG | CGCAGGTGCTGCATGCTG | |  | |
| 10 | Exon 13 | TCCTGGGACAGAGGCTGC | GTCTTCCCATGGTCCTGGC | |  | |
| 11 | Exon 14 | GCTTGGTTCCTTCTGAGACG | GCTGAGGTGCATCTCCTGC | |  | |
| 12 | Exon 15 | CTGTTTCCATGCCGCTTC | ACCTCCCTTCCTCCAGTTTAG | |  | |
| 13 | Exon 16 | TCTTCCGAGGAGCTAAACTGGA | CCAAGGATGGTGATGGCCT | |  | |
| 14 | Exon 17 | CATCCTTGGAGGACTCAAGC | CCACCCAGAGGCAACAGAC | |  | |
| 15 | Exon 18 | GCCTGG TGT GAG CCC TAG AG | AGAGAGGGGCAGAGCAGGT | |  | |
| 16 | Exon 19 | GTGCCTGGCCGAGGG | AAGTCCTCTGCAGCACG | |  | |
| 17 | Exon 20 | TTAGCTGTGCATGGTGGCC | CTGTTTGCGCCCTCGC | |  | |
| 18 | Exon 21 | CTCGCCCTGCATGCCAGCAA | CAATGTTTCCAGGCATGTTTGAT | |  | |
| 19 | Exon 22 | CATGCTCAGGTGGCAGGG | TCCCACCAGACCCAGGG | |  | |
| 20 | Exon 23 | TTGTGCTCTCCCATAAGCCC | AGCTGCCGGCCTAGGG | |  | |
| 21 | Exon 24 | TGAGGACCTCCAAATGCTCC | TGAGCATGAACTGGGCCC | |  | |
| 22 | Exon 25 | CGGATAAGGCCCTGTGCC | GCCCCTGCCCTAGGAGG | |  | |
| 23 | Exon 26 | CTGGCCTTCACATCTCCCC | CTGGCTGTAAGTGCCGCC | |  | |
| 24 | Exon 27 | CTGGGTTCACGCATGC | GCTGACTACCTGGCTGTCC | |  | |
| 25 | Exon 28 | GCATTCCGTGTTTTCTGTTGGG | GGGGTGAGACAGGCATGGC | |  | |
| 26 | Exon 29 | TACACACACTGGCAGGAACC | ACCACATCCCAGCTC | |  | |
| 27 | Exon 30 | CAATTGCTGGGTGTGGGC | CAG TGT GGC TCA CGG GC | |  | |
| 28 | Exon 31 | AGCATGGGTATCAGGAACAGC C | GGCGAGGCACAGACGC | |  | |
| 29 | Exon 32 | GCGTCTGTGCCTCGCC | GGAGTCCTCTGGCAATAGGG | |  | |
| 30 | Exon 33 | CCCTATTGCCAGAGGACTCC | CCCTATTGCCAGAGGACTCC | |  | |
