# Supplementary material for: Heterozygosity for E292V in ABCA3, lung function and COPD in 64,000 individuals
Source: Respir Res. 2012 Aug 6;13(1):67. doi: 10.1186/1465-9921-13-67 (PMC3514156; doi:10.1186/1465-9921-13-67)
Supplement: Additional file 3 — Table S3. Primers and probes for genotyping assays. [file 1465-9921-13-67-S3.doc]

Supplementary table 3. Primers and probes for genotyping assays

| Variation | Method | Forward primer | Reverse primer | VIC-probe | FAM-probe |
| --- | --- | --- | --- | --- | --- |
| H86Y | TaqMan | CCT GGG AGC TTG CCT ACA TC | CCC TGC GCA CTG TCT CA | CGT CAC TGT GAG AAG G | CGT CAC TGT AAG AAG G |
| E292V | TagMan | TGT AAA ACG ACG GCC AGT CAG CAG CGT GAT GGC TTC T | CAG GAA ACA GCT ATG ACC GCG ATG AGG AGG AAG AGG AA | ATG CGC ATG TAC TCC | CAT GCG CAT GTA CAC C |
| P766S | TaqMan | GGT GCC GGC TAT CAC ATG A | GAC CAG CTG GGA GAT GTC TTC | CAG TGC GGC TCC TT | CAG TGC GAC TCC TT |
| S1262G | TagMan | GCT GCC CAA CCA CTG TCT | CTC CGC GTC TCG TAG TTC TC | TAG AAA CTG CTG ACT GCC | AAA CTG CCG ACT GCC |
| R1474W | TaqMan | ATG CCT TGC TGG ACC ACA T | GGA GCC GAG CGT ACA TGA C | CAT CTC CCG GCC TGT C | CAT CTC CCA GCC TGT C |
| A320T | LightScanner | GGG CTC AGC AGC TGG CT | CCA TGC TCA CCT TGA CAC AGA |  |  |
| A1086D | LightScanner | CCT CCA TTG TGG TCT CCA ACT TC | GGG CAG TGC ACA TAC TCG TTA AA |  |  |
