# Supplementary material for: Heterozygosity for E292V in ABCA3, lung function and COPD in 64,000 individuals
Source: Respir Res. 2012 Aug 6;13(1):67. doi: 10.1186/1465-9921-13-67 (PMC3514156; doi:10.1186/1465-9921-13-67)
Supplement: Additional file 4 — Table S4. Genetic variation in the coding regions ofABCA3 in individuals with extreme lung phenotypes in the Copenhagen City Heart Study. [file 1465-9921-13-67-S4.doc]

Supplementary table 4. Genetic variation in the coding regions of *ABCA3* in individuals with extreme lung phenotypes in the Copenhagen City Heart Study

| Gene  region | Nucleotide  substitution | No. of alleles  (allele frequency) | | | | | | Minor allele frequency | Allele frequency  (NCBI SNP database) | AA  residue | Side chain  substitution | Refs. and SNP  ID numbers |
| --- | --- | --- | --- | --- | --- | --- | --- | --- | --- | --- | --- | --- |
|  |  | Asthma  (n=174) | COPD  (n=175) | Interstitial  lung disease  (n=31) | High  FEV1  (n=140) | Low  FEV1 smokers  (n=122) | Low  FEV1  non smokers  (n=118) |  |  |  |  |  |
| Promotor | -1G>A |  |  |  | 1 |  |  | 6.6x10^-4^ |  |  |  |  |
| Exon 5 | 256C>T |  |  |  |  | 1 |  | 6.6x10^-4^ |  | **H86Y** | polar to nonpolar |  |
| Intron 5 | IVS5-50G>A | 1 |  |  |  | 1 | 1 | 0.002 | 0.000 |  |  | rs46725 |
| Intron 5 | IVS5-17A>G | 4 | 9 | 1 | 4 | 4 | 5 | 0.018 | 0.023 |  |  | rs45592133 |
| Exon 6 | 371A>G |  |  |  | 1 |  |  | 6.6x10^-4^ |  | N124S | same |  |
| Exon 6 | 393C>T |  |  |  |  |  | 1 | 6.6x10^-4^ |  | A131A |  |  |
| Exon 6 | 446C>T | 1 |  |  |  |  |  | 6.6x10^-4^ |  | A149V | same |  |
| Intron 6 | IVS6+11G>A | 8 | 12 | 1 | 12 | 7 | 5 | 0.030 |  |  |  |  |
| Intron 6 | IVS6-14C>G | 3 | 2 |  | 10 | 4 | 4 | 0.015 |  |  |  | rs62040683 |
| Intron 7 | IVS7+18G>T |  | 1 |  |  |  |  | 6.6x10^-4^ |  |  |  |  |
| Exon 8 | 681C>T | 4 | 6 | 3 | 8 | 3 | 5 | 0.019 | 0.043 | A227A |  | rs45480502 |
| Exon 8 | 746C>T | 1 |  |  |  |  |  | 6.6x10^-4^ |  | P249L | same | rs139952718 |
| Exon 8 | 824C>T | 1 |  |  |  |  |  | 6.6x10^-4^ |  | A275V | same | rs148221984 |
| Exon 8 | 839G>A |  |  |  |  | 1 |  | 6.6x10^-4^ |  | R280H | same | rs143008553 |
| Exon 8 | 863G>A |  | 1 |  |  |  |  | 6.6x10^-4^ | 0.005 | R288K | same | rs117603931 |
| Exon 9 | 875A>T | 3 | 5 |  |  |  | 1 | 0.006 | 0.007 | **E292V** | polar to nonpolar | rs1499896 |
| Exon 9 | 958G>A | 1 |  |  |  |  |  | 6.6x10^-4^ |  | **A320T** | nonpolar to polar |  |
| Intron 9 | IVS9-20C>A | 30 | 28 | 6 | 29 | 22 | 30 | 0.095 | 0.065 |  |  | rs13332547 |
| Exon 10 | 1059C>T | 30 | 28 | 6 | 32 | 25 | 33 | 0.101 | 0.083 | F353F |  | rs1332514 |
| Intron 11 | IVS11+26T>C | 1 |  |  |  |  |  | 6.6x10^-4^ |  |  |  |  |
| Intron 11 | IVS11-41G>C |  | 1 |  | 1 | 1 |  | 0.002 |  |  |  |  |
| Exon 12 | 1320C>T |  |  |  |  |  | 1 | 6.6x10^-4^ |  | P440P |  |  |
| Exon 12 | 1321G>A | 1 |  |  |  |  |  | 6.6x10^-4^ |  | V441I | same |  |
| Exon12 | 1336G>A |  |  |  |  | 1 |  | 6.6x10^-4^ |  | D446N | same |  |
| Exon 12 | 1457T>A |  |  |  |  |  | 1 | 6.6x10^-4^ |  | F486Y | same |  |
| Exon 12 | 1465A>T |  |  |  |  |  | 1 | 6.6x10^-4^ |  | M489L | same | rs150543946 |
| Intron 12 | IVS12+5G>T |  | 1 |  |  |  |  | 6.6x10^-4^ |  |  |  |  |
| Intron 12 | IVS12+34C>T |  |  |  |  |  | 1 | 6.6x10^-4^ |  |  |  |  |
| Intron 13 | IVS13-37T>C |  |  |  |  |  | 1 | 6.6x10^-4^ |  |  |  |  |
| Intron 14 | IVS14+13C>G |  |  |  |  | 1 |  | 6.6x10^-4^ |  |  |  |  |
| Intron14 | IVS14+33A>G | 264 | 266 | 48 | 214 | 175 | 186 | 0.759* | 0.437 |  |  | rs170447 |
| Exon 15 | 1755C>G |  | 2 |  |  |  | 1 | 0.002 | 0.095 | P585P |  | rs323043 |
| Exon 15 | 1806T>C |  |  |  |  | 1 |  | 6.6x10^-4^ |  | V602V |  |  |
| Exon 16 | 1989C>A | 1 |  |  |  |  |  | 6.6x10^-4^ |  | R663R |  | rs148780137 |
| Intron 17 | IVS17-17G>A | 8 | 9 | 1 | 7 | 8 | 6 | 0.026 | 0.018 |  |  | rs45538638 |
| Exon 18 | 2296C>T | 4 | 2 | 2 | 1 | 1 |  | 0.007 |  | **P766S** | nonpolar to polar | rs45592239 |
| Exon 18 | 2309C>T | 1 |  |  | 2 |  | 1 | 0.003 |  | P770L | same | rs45592239 |
| Intron 19 | IVS19-17T>C |  |  |  | 1 |  |  | 6.6x10^-4^ |  |  |  |  |
| Intron 20 | IVS20+23G>A | 1 |  |  |  |  |  | 6.6x10^-4^ |  |  |  |  |
| Intron 21 | IVS21+17G>A | 1 | 2 |  |  |  |  | 0.002 |  |  |  |  |
| Intron 21 | IVS21+18G>A |  |  | 1 |  |  |  | 6.6x10^-4^ |  |  |  |  |
| Intron 21 | IVS21+34C>T | 197 | 178 | 32 | 130 | 122 | 108 | 0.505* | 0.471 |  |  | rs313909 |
| Exon 22 | 3082T>A |  |  |  | 1 |  |  | 6.6x10^-4^ |  | S1028T | same |  |
| Exon 22 | 3138G>A | 1 |  |  |  |  |  | 6.6x10^-4^ |  | A1046A |  |  |
| Exon 22 | 3257C>A |  |  |  | 1 |  |  | 6.6x10^-4^ |  | **A1086D** | nonpolar to polar |  |
| Intron 22 | IVS22+15C>T |  | 1 |  |  |  |  | 6.6x10^-4^ |  |  |  |  |
| Intron 24 | IVS24+54G>A | 1 |  |  | 1 |  |  | 0.001 |  |  |  |  |
| Exon 25 | 3784A>G | 2 |  |  |  |  |  | 0.001 | 0.003 | **S1262G** | polar to nonpolar | rs35089233 |
| Intron 25 | IVS25+40T>C | 4 | 4 |  | 2 | 1 | 3 | 0.009 |  |  |  |  |
| Exon 27 | 4116C>T | 34 | 39 | 4 | 25 | 27 | 21 | 0.100 | 0.34 | S1372S |  | rs149532 |
| Exon 28 | 4236C>T |  |  |  |  |  | 1 | 6.6x10^-4^ |  | F1412F |  |  |
| Intron 28 | IVS28+35C>T |  | 1 |  |  |  |  | 6.6x10^-4^ |  |  |  |  |
| Exon 29 | 4420C>T | 1 | 2 | 1 | 3 |  | 1 | 0.005 | 0.006 | **R1474W** | polar to nonpolar | rs146709251 |
| Intron 30 | IVS30+23A>G |  | 1 | 1 | 1 | 1 |  | 0.003 | 0.053 |  |  | rs161425 |
| Intron 31 | IVS31+12C>G | 8 | 4 | 2 | 2 | 1 | 1 | 0.012 |  |  |  |  |

Values represent number of individuals (allele frequency). Nucleotide 1 denotes A in the start codon ATG in exon 1 corresponding to base position 713 in mRNA sequence NM_001089. Variants in bold were subsequently screened for in the Copenhagen City Heart Study. * For IVS14+33A>G and IVS21+34C>T minor allele turned out to be the major allele in our population. However, to simplify comparisons to data from the NCBI SNP database we did not change the denotation of the variants. SIFT and Polyphen prediction programs both predicted E292V to be damaging, but none of the other six mutations in bold.
