# Supplementary figures and images for: Heterozygosity for E292V in ABCA3, lung function and COPD in 64,000 individuals
Source: Respir Res. 2012 Aug 6;13(1):67. doi: 10.1186/1465-9921-13-67 (PMC3514156; doi:10.1186/1465-9921-13-67)

## Slide 1
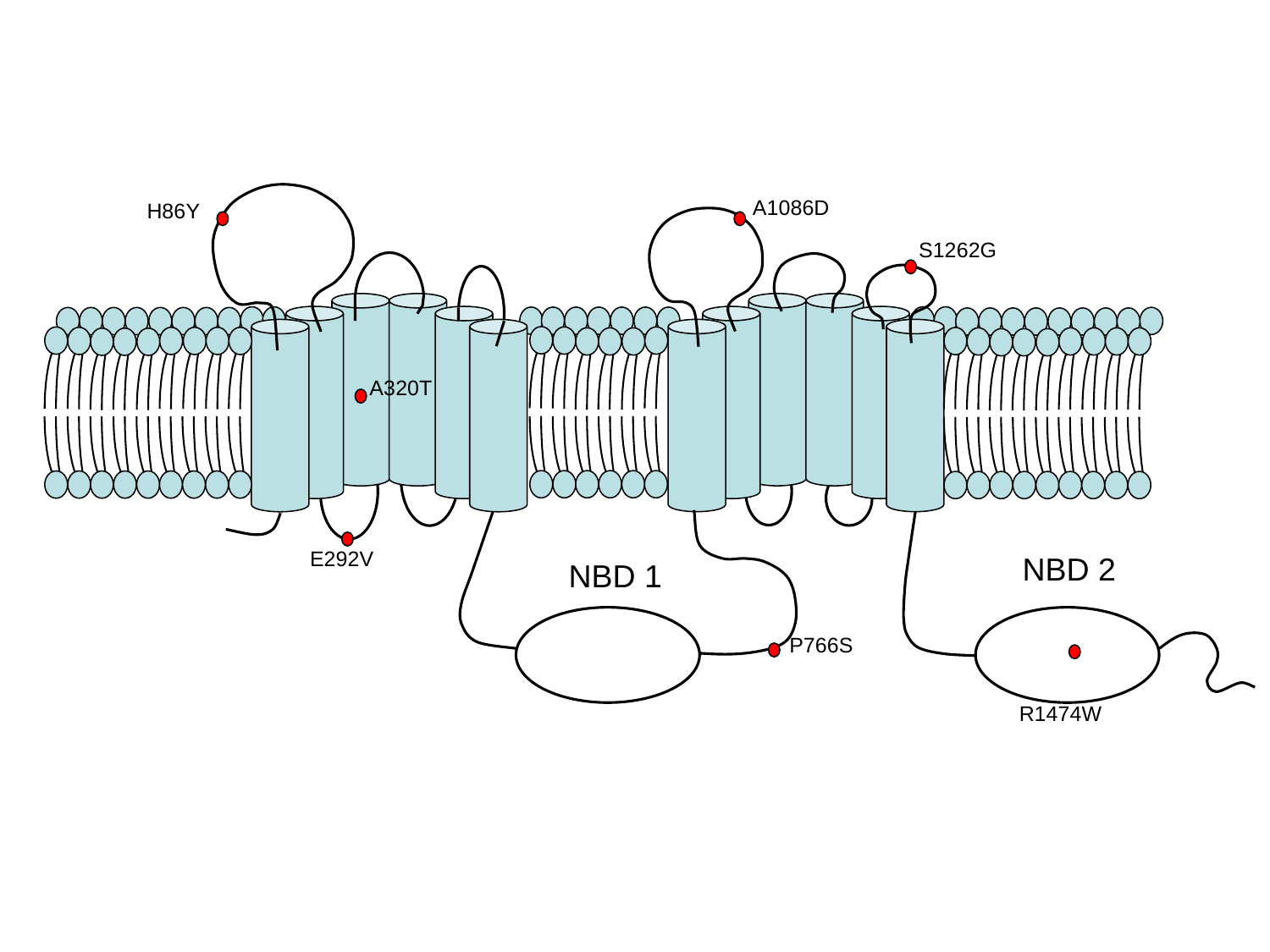

A1086D
H86Y
S1262G
A320T
E292V
NBD 2
NBD 1
P766S
R1474W

Supplement: Additional file 5 — Figure S1. [file 1465-9921-13-67-S5.ppt]

## Slide 1
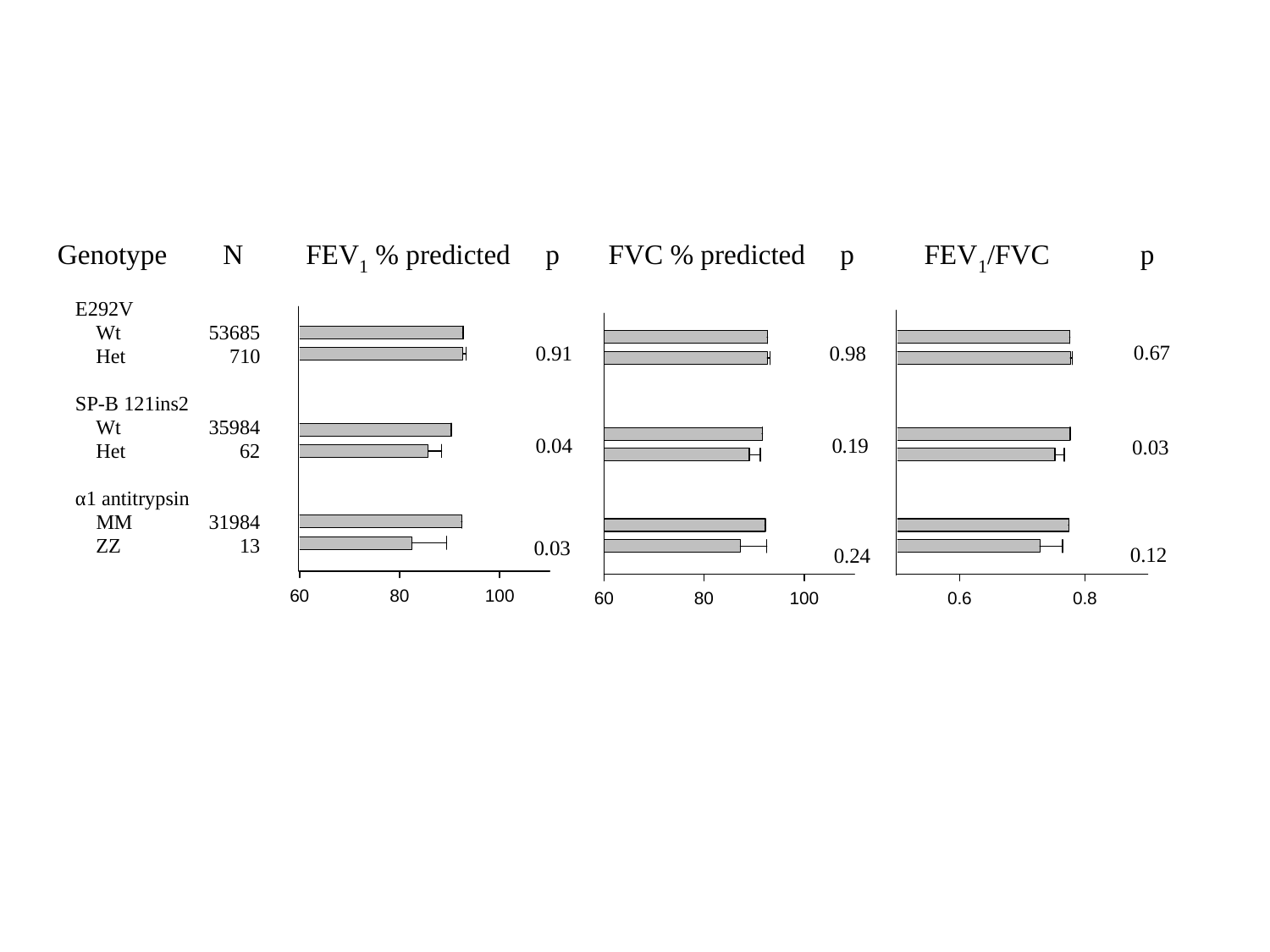

Supplement: Additional file 8 — Figure S2. [file 1465-9921-13-67-S8.ppt]

## Slide 1
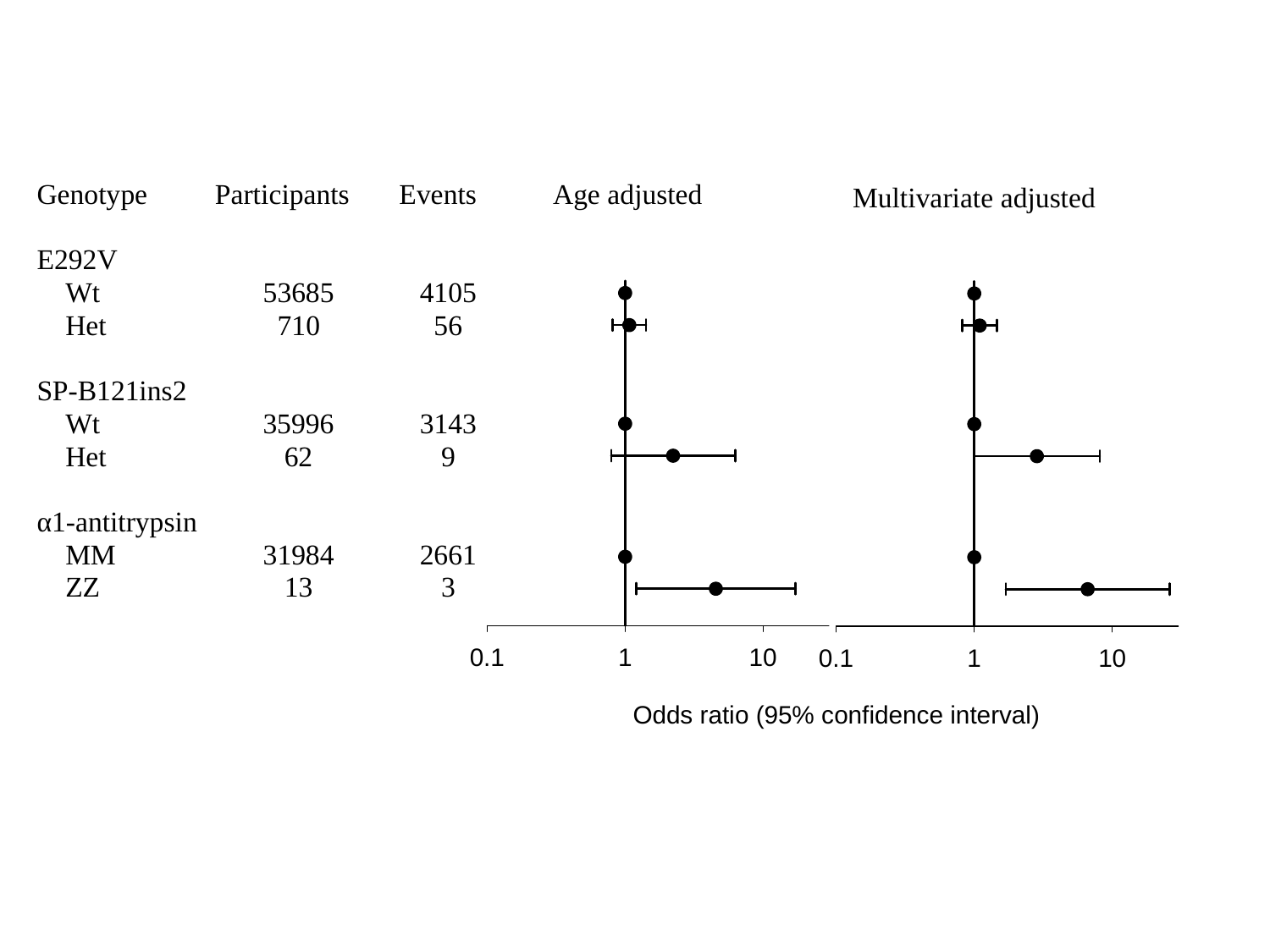

Supplement: Additional file 9 — Figure S3. [file 1465-9921-13-67-S9.ppt]
