# Supplementary material for: Heterozygosity for E292V in ABCA3, lung function and COPD in 64,000 individuals
Source: Respir Res. 2012 Aug 6;13(1):67. doi: 10.1186/1465-9921-13-67 (PMC3514156; doi:10.1186/1465-9921-13-67)
Supplement: Additional file 6 — Table S5. Genotype distribution, minor allele frequency and Hardy-Weinberg statistics for ABCA3 variants identified in the Copenhagen City Heart Study. [file 1465-9921-13-67-S6.doc]

Supplementary table 5. Genotype distribution, minor allele frequency and Hardy-Weinberg statistics for *ABCA3* variants identified in the Copenhagen City Heart Study.

| Genotype | H86Y | E292V | A320T | P766S | A1086D | S1262G | R1474W |
| --- | --- | --- | --- | --- | --- | --- | --- |
| Wildtype | 10,052 | 9,954 | 10,054 | 9,945 | 10,063 | 10,053 | 9,886 |
| Heterozygote | 15 | 113 | 13 | 122 | 4 | 14 | 181 |
| Homozygote | 0 | 0 | 0 | 0 | 0 | 0 | 0 |
| MAF | 0.0007 | 0.006 | 0.0006 | 0.006 | 0.0002 | 0.0007 | 0.009 |
| HW, p-value | 0.94 | 0.57 | 0.95 | 0.54 | 0.98 | 0.94 | 0.36 |

Values represent number, fraction, or p-value. MAF = minor allele frequency. HW = Hardy-Weinberg equilibrium
