# Supplementary material for: Heterozygosity for E292V in ABCA3, lung function and COPD in 64,000 individuals
Source: Respir Res. 2012 Aug 6;13(1):67. doi: 10.1186/1465-9921-13-67 (PMC3514156; doi:10.1186/1465-9921-13-67)
Supplement: Additional file 7 — Table S6. Genotype distribution and minor allele frequency of ABCA3 E292V in the Copenhagen City Heart Study (CCHS) and the Copenhagen General Population Study (CGPS). [file 1465-9921-13-67-S7.doc]

Supplementary table 6. Genotype distribution and minor allele frequency of ABCA3 E292V in the Copenhagen City Heart Study (CCHS) and the Copenhagen General Population Study (CGPS).

|  | CCHS | CGPS | p-value |
| --- | --- | --- | --- |
| Wildtype | 9,954 | 53,685 |  |
| Heterozygote | 113 | 710 |  |
| Homozygote | 0 | 0 |  |
| Minor allele frequency | 0.006 | 0,007 | 0.13 |
